# The development of an online serious game for oral diagnosis and treatment planning: evaluation of knowledge acquisition and retention

**DOI:** 10.1186/s12909-023-04789-x

**Published:** 2023-11-03

**Authors:** Waranun Buajeeb, Jirachaya Chokpipatkun, Napas Achalanan, Nawaphat Kriwattanawong, Kawin Sipiyaruk

**Affiliations:** 1 Department of Oral Medicine and Periodontology, Faculty of Dentistry, Mahidol University, Bangkok, Thailand; 2 Doctor of Dental Surgery Program, Faculty of Dentistry, Mahidol University, Bangkok, Thailand; 3 Department of Orthodontics, Faculty of Dentistry, Mahidol University, Bangkok, Thailand

**Keywords:** Dental education, Serious game, Simulation, Oral diagnosis, Oral lesion

## Abstract

**Background:**

While serious games seem to be supportive in healthcare education, none of them had been designed to develop competence in diagnosis and treatment planning of oral lesions. Therefore, this research aimed to develop an online simulation-based serious game for training diagnosis and treatment planning of oral lesions (SimOL) and to evaluate its educational impact in terms of knowledge improvement and retention.

**Methods:**

As a mandatory task in an oral lesion course, all 28 students were required to participate in SimOL activities. Participants were instructed to complete a pre-knowledge assessment following a one-week washout period prior to the game activity. Subsequent to the game completion, they were tasked to complete a post-knowledge assessment I (Full score = 15) and satisfaction questionnaire. A post-knowledge assessment II was administered a week later to evaluate knowledge retention.

**Results:**

The findings demonstrated a significant increase in the assessment scores after interacting with the game (*P* < 0.001), where the pre- and immediate post-knowledge assessment scores were 8.00 (SD = 2.11) and 11.71 (SD = 2.39), respectively. The game also exhibited a positive impact on knowledge retention, as there was no significant difference between the scores of post-knowledge assessment I and II (*P* > 0.05). Additionally, students perceived the game as positively in all aspects, although the entertainment aspect achieved a slightly lower score of 3.70 (SD = 0.21), in comparison to the usefulness and ease of use with a score of 4.02 (SD = 0.11) and 4.02 (SD = 0.16), respectively.

**Conclusion:**

SimOL demonstrated its potential as an effective learning tool for improving and retaining knowledge for diagnosis and treatment planning of oral lesions. The game was perceived positively by dental students in all aspects, however further improvements should prioritize the enhancement of entertaining components.

## Introduction

The new generation of learners appear to be familiar with technologies including video games. Their characteristics and learning behaviors have changed due to their daily exposure to technology, known as ‘digital natives’ [1]. Instead of traditional teaching strategies, they tend to prefer technology-enhanced learning (TEL), as it allows instructors and learners to have flexibility in terms of time, location, and pace [2, 3]. In combination with gaming features, TEL can be strategically designed and developed to be more engaging known as serious games.

Serious games have gained increasing recognition as effective TEL in healthcare areas [4–6], including dental education [7]. Existing evidence in dental education indicates that learners could gain knowledge after interacting with serious games [8–10]. Serious gaming technology also enables an activity log function to indirectly observe how learners interact with the game [11]. This function allows instructors to detect students who have learning misbehaviors and support them to achieve expected learning outcomes. Therefore, serious games should be further developed and implemented in various disciplines of dental education, including dental medicine.

Oral medicine is a dental specialty purposing to diagnose and manage patients with oral mucosal lesions. Competence in oral medicine has been considered as a prerequisite at both dental undergraduate and postgraduate levels although there appear to be heterogeneities of oral medicine practice due to geographical variations [12]. As emotional intelligence and clinical decision making are considered as important [13], clinical training is required to achieve expected competence in diagnosis and management of oral lesions, rather than relying solely on classroom settings [14, 15]. Hence, the development of simulation-based serious games in oral medicine should be recommended for students to apply their knowledge in virtual patients before commencing clinical training.

While clinical reasoning and diagnostic skills can be developed through gamified virtual patient experiences [16–18], there has been no serious game designed specifically to develop competence in diagnosis and treatment planning of oral lesions. Consequently, this research was conducted to develop an online simulation-based serious game for training dental undergraduates in the diagnosis and treatment planning of oral lesions, as well as to evaluate its educational impact in terms of knowledge improvement and retention along with user satisfaction.

## Materials and methods

### An online serious game in diagnosis and treatment planning of oral lesions

An online simulation-based serious game for the diagnosis and treatment planning of oral lesions (SimOL) was designed and developed in a simulation-based format, where learners could be exposed to virtual patients with orofacial problems. The game was developed using ‘React’ which was an open-source JavaScript framework and made available on both iOS and Android platforms.

**Fig. 1:**
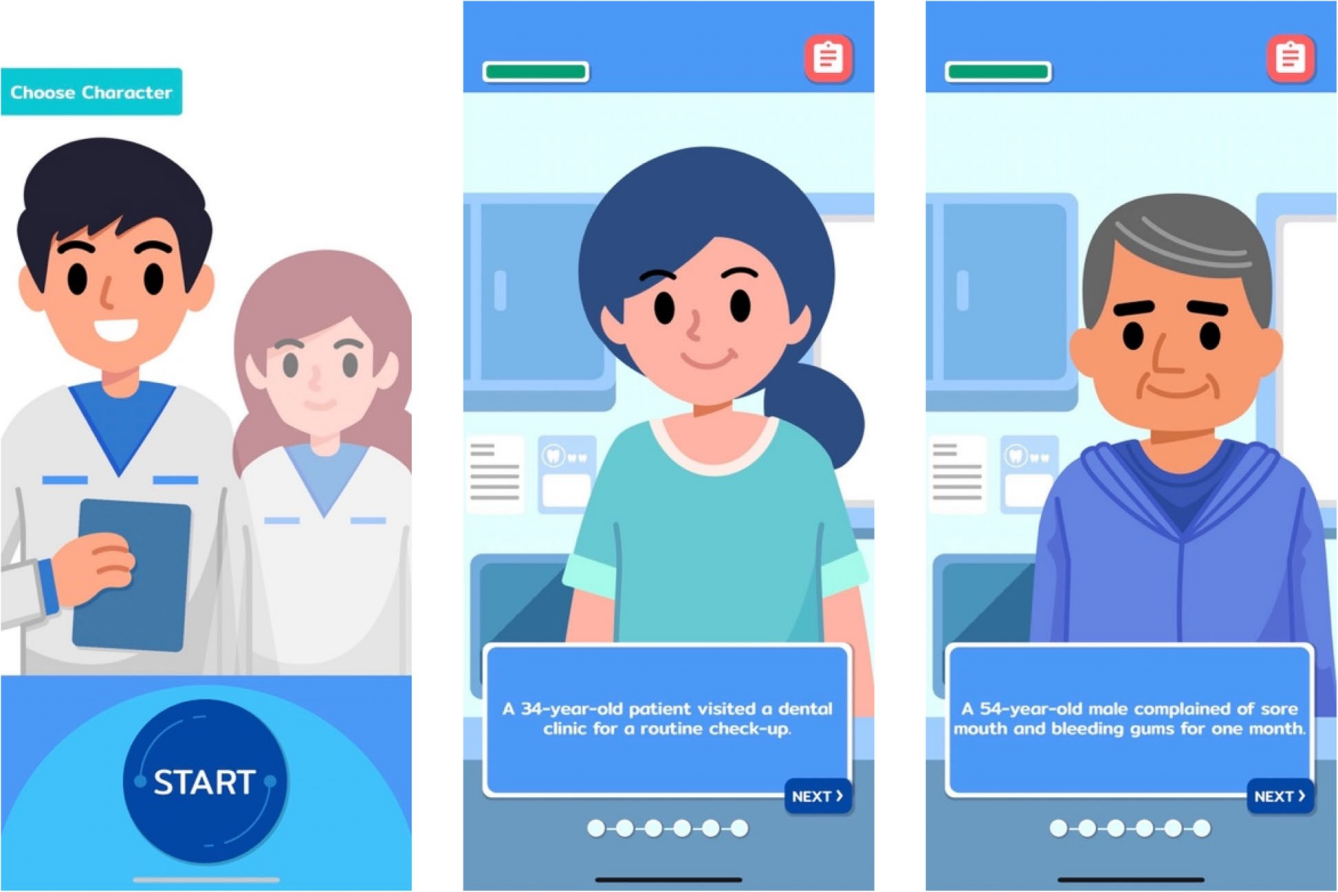
User interface of SimOL where learners could interact with the game

Following a registration or log-in process, learners were provided the option to select their in-game character, choosing from either a male or female dental student. Subsequently, they could proceed to select one of the three virtual patients (learning scenarios) where they could be exposed to various oral lesions, including mucous membrane pemphigoid, allergic contact stomatitis, and pemphigus vulgaris. Within each learning scenario, students were initially provided with a chief complaint and oral features. They were subsequently directed to collect additional information, such as present illness and medical history of a simulated patient, to provide differential diagnosis. Following this, students were tasked with the selection of appropriate options for laboratory investigations, such as direct/indirect immunofluorescence and histopathology, to gather further information about the oral lesion. They were finally required to provide a definite diagnosis and develop a treatment plan for the patient. The user interface where learners could interact with the game are presented in Fig. 1.

**Fig. 2:**
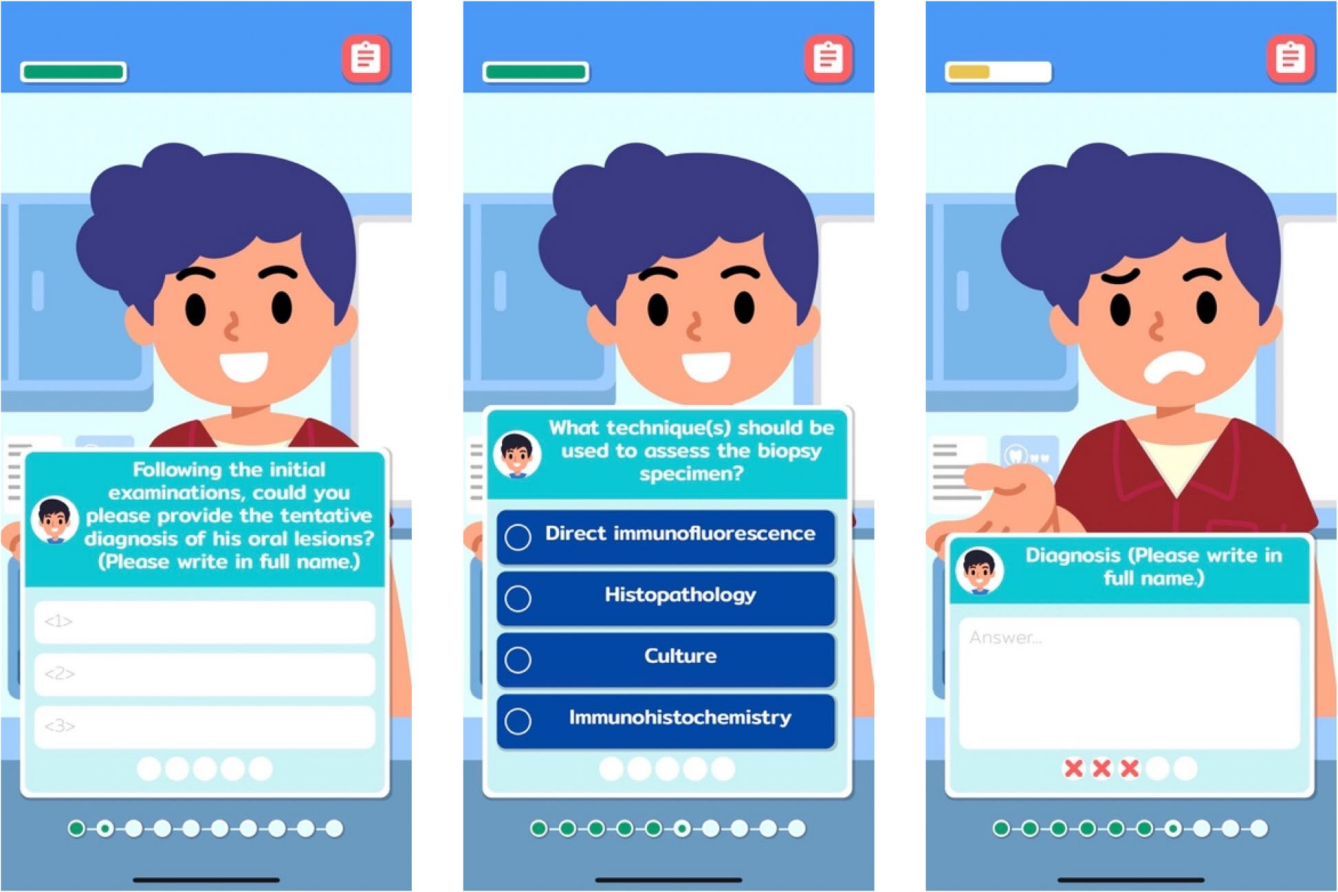
Examples of question formats provided in the game

One key feature of SimOL was the incorporation of diverse interactive quiz formats within the game, which were designed as application-level learning with an expectation of knowledge retention. In addition to single and multiple response questions, an open-ended format was designed for questions regarding differential diagnosis. This format enables learners to consider gathered patient information and freely offer a definite diagnosis and treatment planning to the game system similar to clinical situations, where predetermined options are not available. The design and implementation of the open-ended question format is considerably more complicated, compared to a closed-ended format. As users can freely respond to the game, the development team was tasked to carefully construct the answer pool of each question covering all possible sets of correct answers. For example, ‘desquamative gingivitis’, ‘gingival ulceration’, ‘gingival ulcer’, and ‘gingival erythema’ were required for the answer pool of a question regarding clinical features of gingival tissue. Moreover, ‘mucous membrane pemphigoid’, ‘mucous membrane pemphigoid (MMP)’, and ‘MMP’ were required for a question regarding a definite diagnosis of one virtual patient. This will assure that students can get a score even though they do not provide an exact answer. Examples of question formats provided in SimOL are presented in Fig. 2.

**Fig. 3:**
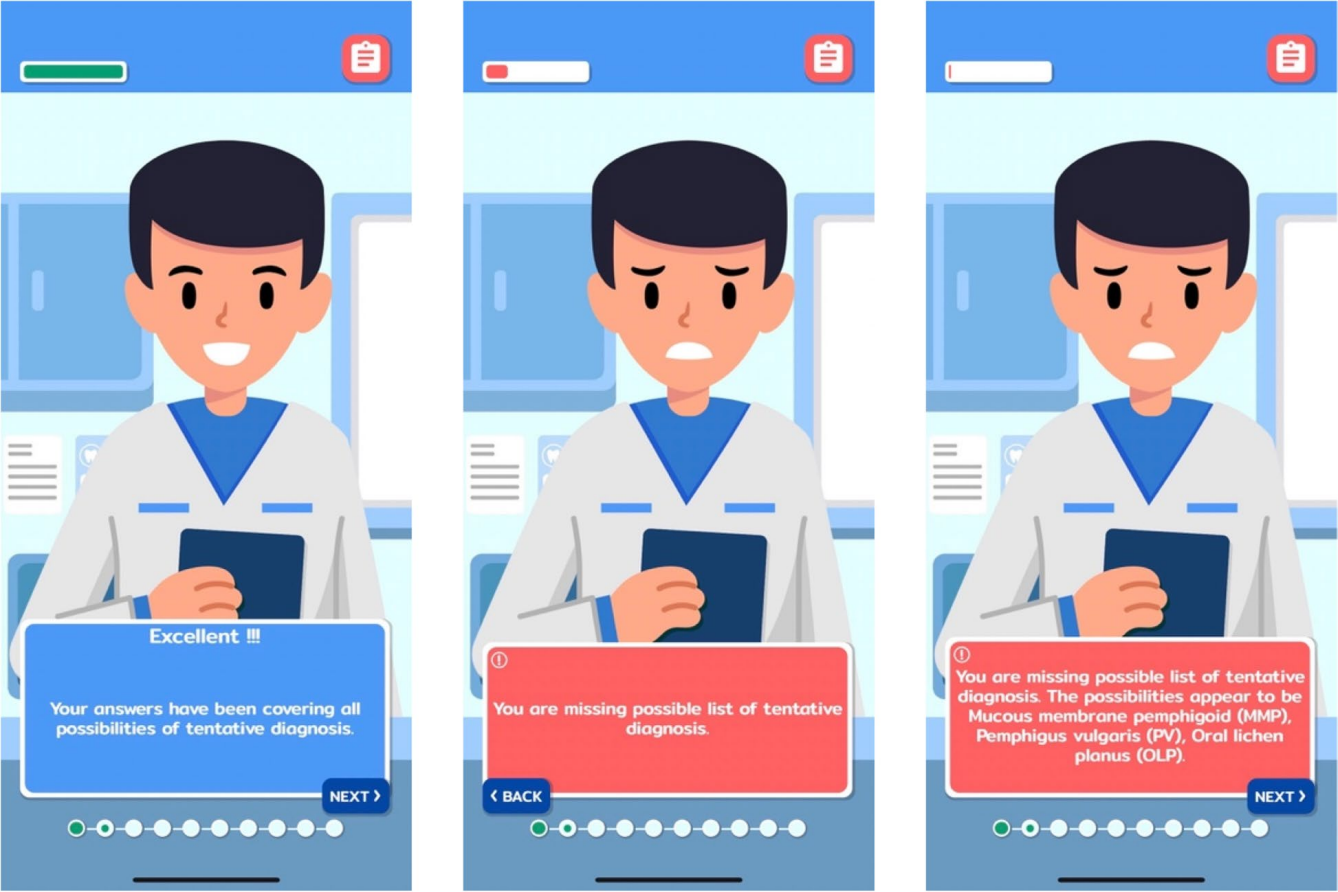
Examples of feedback formats provided within the game

According to the feedback system within the game, learners could receive immediate responses in various formats following their interactions. There was informative feedback delivered by a virtual instructor, explaining clinical reasoning behind each question, regardless of whether answers were correct. Clues were also offered when incorrect responses were selected. Furthermore, when learners chose a wrong answer, their score would be deducted and represented by facial expression of the virtual patient. Examples of feedback formats provided within the game are shown in Fig. 3.

### Research design

This study employed a quantitative research approach, using a pre-experimental design. SimOL was used as mandatory of an oral lesion course for third year dental undergraduates from the Mahidol International Dental School, Faculty of Dentistry, Mahidol University. All students were required to complete a pre-knowledge assessment to investigate their background knowledge. After a one-week washout period, the students were allowed to interact with SimOL, where they had three hours to complete three learning scenarios of the game. They were then immediately asked to complete a post-knowledge assessment I and a self-reflection report on their learning to evaluate knowledge gain achieved from the game tasks. A post-knowledge assessment II was administered a week later to evaluate knowledge retention. In addition, a satisfaction questionnaire was distributed to students to explore their perceptions toward the game. All students had to complete all assigned tasks independently without discussion with their peers. These learning activities were conducted in January 2023.

### Research participants

As SimOL was a requirement of course completion, all 28 third year dental undergraduates from the Doctor of Dental Surgery Program (International Program) in academic year 2022 had participated in this research. They also completed all assigned tasks at the same time to minimize the contamination of knowledge assessment.

### Outcome measurements

#### Knowledge assessments

To evaluate their knowledge improvement, all students were required to complete pre- and immediate post-knowledge assessments, which could be considered as the most common approach for the serious game evaluation [7, 19]. In addition, knowledge retention was assessed in this research using post-knowledge assessments I and II. All assessments had 15 similar multiple-choice questions, so the total score was 15 marks. However, the questions and answers choices of all three assessments were randomly arranged to reduce the test–retest memory effect [10, 20–22]. The knowledge assessment was firstly developed by a member of research team, who was an expert in oral medicine (W.B.). The content of SimOL and assessment were then evaluated by other three experts in oral medicine who had no contribution in this study using content validity to confirm its suitability in achieving the expected learning outcomes of the game. The reliability of the assessment was also evaluated using Kuder-Richardson Formula 20 (KR-20).

#### User satisfaction

The paper-based questionnaire was developed using the technology-acceptance model to gather user satisfaction [23], where the questions were adapted from previous literature [10, 22]. The satisfaction questionnaire comprised 15 five-point Likert scale questions, covering ‘perceived usefulness’ (5 items), ‘perceived ease of use’ (5 items), and ‘perceived enjoyment’ (5 items), in which ‘1’ being ‘Strongly disagree’ and ‘5’ being ‘Strongly agree’. Two open-ended questions were also included to explore strengths and weaknesses of the game.

To assure the quality of the satisfaction questionnaire, content validity was assessed by three experts in dental education, and the questions were iteratively revised until the index of item-objective congruence of each item was higher than 0.5. In terms of reliability, the questionnaire was piloted and evaluated by considering internal consistency. Problematic items were revised or removed until the Cronbach's alpha coefficients of all constructs higher than 0.7 (Perceived usefulness: 0.81; Perceived ease of use: 0.84; Perceived enjoyment: 0.85).

### Data analysis

The research data were analyzed using the Statistical Package for Social Sciences software (SPSS, version 28, IBM Corp., Armonk, NY). Descriptive statistics were performed to present an overview of the data. Knowledge improvement and retention were analyzed using a repeated measures ANOVA with the Bonferroni post-hoc test, where the significance level was set at *P* < 0.05. The quantitative content analysis was also performed to quantify qualitative data from the open-ended questions.

### Ethical considerations

This research protocol was approved by the Institutional Review Board of Faculty of Dentistry and Faculty of Pharmacy, Mahidol University on 29 November 2022 (the certificate of exemption number: MU-DT/PY-IRB 2022/054.2911).

## Results

### Reliability assessments of the data collection tools

The research data retrieved from the three knowledge assessments and satisfaction questionnaire were assessed to confirm their reliability even though they were piloted and evaluated prior to the data collection. KR-20 of the pre-knowledge assessment was 0.54, while those of post-knowledge assessment I and II achieved higher values, which were 0.68 and 0.65, respectively. The internal consistency of the satisfaction questionnaire was also assessed with Cronbach's alpha (Perceived usefulness: 0.92; Perceived ease of use: 0.94; Perceived enjoyment: 0.94).

### Knowledge improvement and retention

The results retrieved from the repeated measure ANOVA analysis demonstrated a statistically significant difference of the scores among those three knowledge assessments [F(1.43, 38.49) = 27.31, *P* < 0.001], as presented in Table 1.

**Table 1:** Repeated measure ANOVA of the scores from the three knowledge assessments

Source	Sum of Squares	df	Mean square	F	*P*-value
Assessment scores	265.17	1.43	186.00	27.31	< 0.001
Error	262.17	38.49	6.81		

The degrees of freedom were adjusted using Greenhouse–Geisser estimates of sphericity (ε = 0.71)

The Bonferroni post-hoc test revealed that there was an improvement of the knowledge assessment scores among dental undergraduates after interacting with the game (*P* < 0.001). The assessment score increased from 8.00 (SD = 2.11) for the pre-knowledge assessment to 11.71 (SD = 2.39) for the post-knowledge assessment I. According to knowledge retention, the score of 11.82 (SD = 1.91) for the post-knowledge assessment II showed no statistically significant difference when compared to the post-knowledge assessment I (*P* > 0.05). These findings were presented in Table 2.

**Table 2:** Bonferroni post hoc test for pairwise comparisons

Source	Mean (SD)	Comparison	Mean difference (SD)	*P*-value
Pre-assessment	8.00 (2.11)	Post-assessment I Post-assessment II	-3.71 (3.74) -3.82 (3.38)	< 0.001 < 0.001
Post-assessment I	11.71 (2.39)	Pre-assessment Post-assessment II	3.71 (3.74) -0.11 (1.93)	< 0.001 1.000
Post-assessment II	11.82 (1.91)	Pre-assessment Post-assessment I	-3.82 (3.38) 0.11 (1.93)	< 0.001 1.000

### User satisfaction

The satisfaction questionnaire was completed and returned by all research participants. The findings indicated that the students tended to perceive the game as positively. ‘Perceived usefulness’ and ‘Perceived ease of use’ appeared to be rated as mostly positive by the research participants with the score of 4.02 out of 5 (SD = 0.11) and 4.02 out of 5 (SD = 0.16), respectively. ‘Perceived enjoyment’ was considered as least positive with the score of 3.70 out of 5 (SD = 0.21). The findings retrieved from the satisfaction questionnaire were presented in Table 3.

**Table 3:** User satisfaction retrieved from the satisfaction questionnaire

Satisfaction	Mean (± SD)
Perceived usefulness	4.02 (0.11)
Perceived ease of use	4.02 (0.16)
Perceived enjoyment	3.70 (0.21)

There were 22 students who provided their responses to the open-ended questions (Table 4). Eight participants supported the use of game format, as they felt engaged with the learning activity. Visualization, virtual patients, and immediate feedback were raised by a couple of participants, as these features of the game were engaging and supporting them to learn. The navigation was also perceived as straightforward. However, there were eight negative responses. Open-ended questions within the game seemed to be perceived as problematic, as exact spellings were required and just a typo would be considered as incorrect. Two students responded that they were annoyed when the cartoon characters were hidden by the pop-up keyboard during answering the open-ended questions.

**Table 4:** Qualitative responses toward the gameplay retrieved from the open-ended questions

Category	Code	Example	Frequency
Supporting features	Game format	The game format allowed me to be engaged with the learning activity.	8
	Visualization	The cartoon characters were lovely, and the graphics can make learning content easy to understand.	5
	Virtual patient	The virtual patients could simulate training situations, like I can practice in a clinic.	4
	Immediate feedback	The feedback provided instantly within the game helped me to learn.	4
	Navigation	The navigation was straightforward.	1
Challenges	Open-ended question format	The open-ended questions required only the exact spellings.	6
	User interface when typing an answer	It was annoying that the pop-up keyboard always hid the cartoon characters.	2

## Discussion

SimOL demonstrated its potential as an effective learning tool for training diagnosis and treatment planning of oral lesions, as evidenced by the findings retrieved from knowledge assessments. This trend was consistent to results from other studies evaluating serious games in dental education [7]. Students also perceived SimOL as supportive for their learning according to its gaming features. Within the serious game, students could gain knowledge from the role of failure [24, 25]. SimOL provided immediate feedback in various formats, allowing students to learn from their mistakes. In other words, students could modify their responses or strategies until they complete the game tasks. Therefore, learners can improve their competencies through engagement with serious gaming activities.

In addition to fostering knowledge improvement, SimOL exhibited positive impact in terms of knowledge retention. The findings revealed no statistically significant difference between the post-knowledge assessment I and II, even though there were no learning activities in diagnosis and treatment planning of oral lesions during that period. This positive effect on knowledge retention has also been observed in previous studies [26–28]. However, there appears to be variability in the assessment of knowledge retention, spanning durations of follow-up periods from 24 h to 18 months [29–33]. Consequently, SimOL can be considered as having a positive impact on at least short-term knowledge retention (one-week follow-up period), as the game enabled students to engage in critical thinking and practical application of their knowledge within simulated clinical scenarios.

The navigation and usability of serious games can be considered as important. A complicated navigation could distract students from serious gaming activities, which could have negative impact on the achievement of learning outcomes [10]. SimOL was perceived by students as user-friendly, as its navigation was straightforward. They were able to perform the game tasks without the usability interfering their learning. It could be a result from the design of SimOL in a mobile application which was applicable for both iOS and Android smartphones, where these students had already been familiar with.

Engagement could be considered as an area of potential improvement of SimOL. A number of entertaining features were incorporated into the game, including graphics (cartoon characters and user interface), audio elements, storytelling, achievement system, and a degree of freedom. These features can be considered as the key components of serious game design [10, 34]. However, perceived enjoyment was rated by students with the lowest score compared to other aspects. Enjoyment seems to be challenging for serious game design, as there should be a balance between learn and play [35]. Social interaction is another feature to be considered, as there is evidence that collaboration or competition between users could have impact on perceived usefulness and enjoyment [10]. A comprehensive approach to incorporating these features is essential to enhance the entertaining aspect of SimOL.

The level of difficulty is a crucial consideration, as it has the potential to influence the flow of the game. According to the flow theory, there should be a balance between game challenges and user competencies [36]. Users can become disengaged if a serious game is too simplistic, however they could feel frustrated if a game task is too challenging [37]. According to student feedback on SimOL, the open-ended questions were perceived as particularly challenging due to the requirement for precise answers, and they would not receive a score even minor spelling errors. Furthermore, students found it challenging to provide or guess a potential answer if they lacked the necessary knowledge. The next iteration of the game may be designed with different levels of difficulty for each learning scenario, allowing students to select a challenge matching to their level of competence. For instance, there would be a list of answer options provided for students at a basic level or when they fail to complete a game task several times. This approach would enhance the feasibility and practicality of open-ended questions in serious games.

 This research was carefully designed to minimize research biases, particularly the test–retest memory effect, including the wash-out period and the random sequences of questions and answers choices. The data collection tools were also developed and evaluated to assure their validity and reliability, thereby enhancing the research quality. However, there were limitations of this research. Since SimOL was a mandatory activity in the oral lesion course, all students were required to complete the game tasks. Therefore, a randomized control trial could not be conducted to compare the educational impact between SimOL and other traditional approaches. Moreover, students were mandated to complete all tasks (SimOL and knowledge assessments) simultaneously in a classroom to minimize possible confounding factors, and therefore it was not feasible to assess actual learning behaviors. The limited number of students participating in this study appeared to be another constraint, so further evaluation of the game with a larger and more diverse cohort of learners should be considered. Consequently, further research should be required to confirm the effectiveness of SimOL and to investigate actual learning behaviors of students through the activity log function.

## Conclusions

SimOL appears to be supportive as an interactive learning tool in training diagnosis and treatment planning of oral lesions, according to its positive educational impact in terms of knowledge acquisition and retention, as evidenced in this research. The findings also revealed several areas of improvement, which could be applied for the next iteration of SimOL and other serious games. However, further research should be required to confirm its effectiveness and feasibility in dental education.

## Data Availability

The data that support the findings of this study are available from the corresponding author, up-on reasonable request. The data are not publicly available due to information that could compromise the privacy of research participants.
